# Opioid-Associated Amnestic Syndrome

**DOI:** 10.7759/cureus.16714

**Published:** 2021-07-29

**Authors:** William Ciurylo, Elizabeth Noh

**Affiliations:** 1 Internal Medicine, Portsmouth Regional Hospital, Portsmouth, USA; 2 Internal Medicine, Tufts Medical Center, Boston, USA

**Keywords:** opioid, fentanyl, amnesia, hippocampus, opioid associated amnestic syndrome

## Abstract

A 31-year-old male with a history significant for obesity, attention deficit hyperactivity disorder, methamphetamine use, and IV drug use was evaluated for unexplained global amnesia greater than 24 h. The patient had been in recovery for opioid use disorder for about a year, but he relapsed on IV fentanyl in the week prior to presentation. On exam, he was alert and fully oriented but had no spontaneous recall of three objects after five minutes. General medical and neurological examinations were otherwise unrevealing. Urine fentanyl and norfentanyl were positive. CT and MRI imaging demonstrated isolated bilateral hippocampal injury. Given the totality of his presentation and the contributing variables, his medical team considered this to be a case of the newly characterized opioid-associated amnestic syndrome (OAS). This case is significant because of the relative absence of potentially confounding variables on presentation, including antecedent cardiorespiratory failure. Further reporting of these cases may have implications for understanding opioid toxicity and clarifying the functional role of the hippocampus.

## Introduction

A cluster of 18 cases of opioid-associated amnestic syndrome (OAS) from Massachusetts between 2012 and 2018 was defined by amnesia with bilateral hippocampal injury in association with opioid use [1-3]. A total of 40 confirmed, probable, or possible cases were identified on a subsequent systematic review; the cohort was multi-national and included iatrogenic opioid use as well. By the authors’ criteria, all the cases were characterized by amnesia for greater than 24 h duration and were considered confirmed if there were positive toxicology for opioid and bilateral hippocampal injury on brain imaging; probable if there were known history of opioid use and the mentioned imaging finding; possible if there was one of the following: positive toxicology for an opioid, known history of opioid use, or imaging finding. Based on the case definition, 20 of their 40 cases were considered confirmed [1]. The purpose of this study is to report an additional confirmed case of OAS.

## Case presentation

A 31-year-old male with a history significant for obesity (body mass index, BMI 32.8 kg/m2), attention deficit hyperactivity disorder, as well as methamphetamine and IV drug use (opioids including fentanyl) was transferred from an outside hospital emergency department (ED) for further evaluation of unexplained global amnesia. The patient admitted that he had no recollection of the past few days. A detailed review of systems was otherwise negative. He reported a history of an opioid use disorder for which he had been in recovery for about a year. However, after the recent overdose death of his roommate, he relapsed with what he believed was IV fentanyl in the week prior to presentation. Other history included alcohol and tobacco use, and more recently vaping. On exam, he was alert and fully oriented and could name the previous presidents back to Reagan but had no spontaneous recall of three objects after five minutes. He was able to retrieve all three with cues. General medical and neurological examinations were otherwise unrevealing.

**Figure 1:**
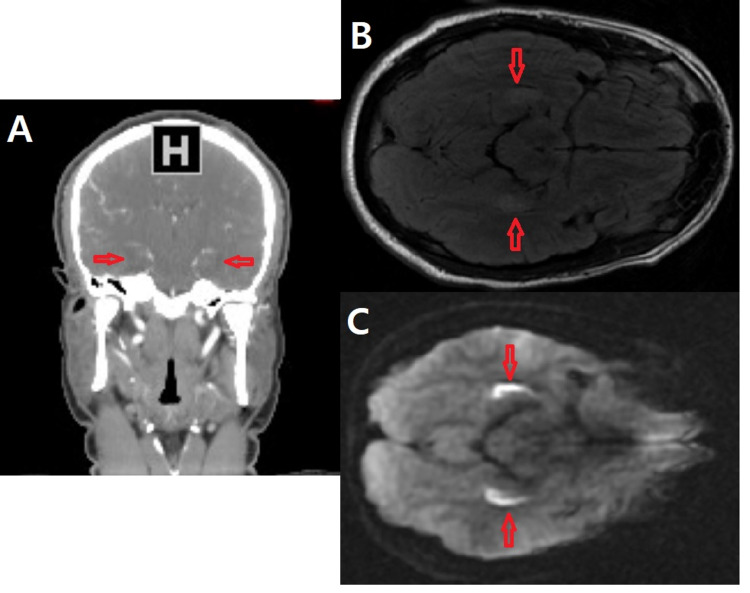
Cerebral imaging studies of a patient presenting with dense anterograde amnesia, with short interval retrograde amnesia, and concomitant IV fentanyl use. A. CTA (coronal view) demonstrates relative hypoattenuation along the bilateral hippocampi B. MRI FLAIR (axial view) demonstrates high signal intensity along the bilateral hippocampi C. Diffusion-weighted MRI (axial view) demonstrates high signal along the bilateral hippocampi correlating with restricted diffusion (ADC image not shown) CTA, CT angiography; FLAIR, fluid-attenuated inversion recovery

CT of the head demonstrated hypoattenuation along the hippocampi bilaterally, while CT angiography (CTA) of the head and neck was unrevealing. On MRI, fluid-attenuated inversion recovery (FLAIR) demonstrated high signal intensity along the bilateral hippocampi with associated restricted diffusion (Figure 1). Such a finding on imaging is suggestive of an ischemic stroke, prompting a full stroke work-up.

As part of this more focused work-up, additional lab work was ordered. Urinalysis was found normal, but urine toxicology demonstrated the presence of urine fentanyl (0.2 ng/mL) and urine norfentanyl (23.3 ng/mL). Urine testing for sufentanil and alfentanil was negative. Other labs, including complete blood count, complete metabolic panel, thyroid stimulation hormone, ammonia, sedimentation rate, C-reactive protein, and antinuclear antibodies were unrevealing. Hypercoagulability workup was unremarkable. Vitamin B12 was 209 pg/mL and folate was 4.6 ng/mL. Vitamin B12, folate, and thiamine were administered during his hospital course without improvement in mentation.

Routine electroencephalogram at presentation was a normal waking and sleep recording with no clear focal or epileptiform abnormalities suggestive of seizure activity. Transthoracic and transesophageal echocardiography were without abnormalities, including atrial septal defects. Holter monitoring study post-hospitalization was also unremarkable.

The hospital course was stable, with no significant regain of function over the duration. The patient had to record details that he referred to orient himself to his conditions. After three days of hospitalization, the patient was discharged into the care of his parents, with a significant disability. He did not remember what happened to him, was confused by his diagnosis and prognosis, and had begun to write a daily log to support his memory. Treatment included aspirin and clopidogrel for 90 days, followed by aspirin monotherapy thereafter. He was discharged with B12 and folate supplementation. 

## Discussion

This patient demonstrated new-onset amnesia of duration greater than 24 h and bilateral hippocampal injury in the setting of confirmed opiate use. After an extensive workup to exclude alternative etiologies, he was diagnosed with the newly characterized OAS [1].

The mechanism by which opiates, particularly fentanyl, can result in bilateral hippocampal injury is unclear. Rat models suggest that fentanyl may have a toxic effect on the hippocampus and limbic system that may result in increased metabolic demand or epileptiform activity [4-5]. In particular, stimulation of hippocampal mu receptors by opiates may inhibit gamma aminobutyric acid (GABA)ergic inhibitory interneurons, resulting in disinhibition of excitatory neurons, epileptiform activity, hypermetabolism, and neural apoptosis. Excessive metabolic demand may be compounded by hypoxemia in the setting of drug-induced hypoventilation [2]. Relative hypoglycemia may be a factor as well [6]. Both epileptic activity and hypoglycemia can present as restricted diffusion in the deep gray matter on diffusion-weighted imaging [7].

This present case is notable for an absence of identifiable confounding features on presentation. Our patient was alert and ambulatory without evidence of cardiorespiratory failure on arrival. The patient also had no prior history of amnestic episodes or seizures. In the initial case series from Massachusetts, only five of the 14 patients identified were without known antecedent loss of consciousness or arrest [3]. In addition, nine of the 14 demonstrated extra-hippocampal signal abnormalities on imaging, while five of the eight with positive opiate toxicology also tested positive for other substances including cocaine, amphetamines, cannabinoids, and benzodiazepines.

It should also be noted that lone cocaine use and carbon monoxide poisoning have each been implicated in mediating a similar pattern of injury [3, 8-9]. Thus, the absence of a reported history or toxicological testing supportive of opioid use could reflect either an incomplete evaluation for opioid use or, in select cases, an alternative etiology altogether [1]. These possibilities underscore the importance of performing confirmatory testing specifically for fentanyl, its analogs, and metabolites in the diagnosis of OAS.

## Conclusions

In the current case, prolonged amnesia associated with isolated hippocampal injury on imaging and an opioid as the only substance present on toxicology screening all strongly point to an amnestic syndrome secondary to opioid-related injury of the hippocampus. The characterization of additional cases and further exploration of the effects of opioids on the hippocampus are necessary to clarify the pathophysiology of OAS and to understand the variable duration of the amnesia thus far observed. Additional follow-up and therapeutic studies may help to determine prognosis and whether potential interventions, such as cognitive rehabilitation, may have restorative benefit. Further reporting of these cases may have implications for understanding opioid toxicity and clarifying the functional role of the hippocampus.
